# Urea in Its Relations to Animal Physiology

**Published:** 1854-11

**Authors:** Thomas Bischoff


### UREA IN ITS RELATIONS TO THE GENERAL PHENOMENA OF ANIMAL PHYSIOLOGY.

BY THOMAS BISCHOFF.

There is no longer any doubt that accurate knowledge of the phenomena or animal organism can only be acquired by the aid of a more intimate acquaintance with the unceasing chemical metamorphoses which take place in them. These changes must be understood, not only qualitatively but quantitatively, before our views on this subject can possess any scientific precision.

Towards the attainment of this object much has already been achieved, but indefinitely more remains to be done. This is particularly the case with regard to the metamorphoses of the nitrogenous constituents of the organism which are justly considered to be of such predominant importance in its actual activity. The history of the nitrogenous elements of food is at the present time incomparably more extended and minute than formerly. So likewise the study of the nitrogenous excretions, particularly urea, has been abundantly and productively cultivated. No doubt is entertained that it is derived from the nitrogenous elements of food, but with regard to the quantitative and even qualitative relations which obtain between them there is the greatest uncertainty and diversity of opinion.

While some regard urea as the ultimate product of a series of metamorphoses of the nitrogenous elements of food which can be developed only in the living organism and by the action of the organs, others entertain the opinion that the albumen of the blood is converted directly into urea, even in the blood.

According to the former view, urea, independently of some other less important nitrogenous excretions, might be regarded as a quantitative measure of metamorphoses in the nitrogenous organs, a circumstance which would be of inculculable value with reference to the functions and agency of these organs. Such a proceeding is, however, inconsistent with the latter view, which represents the quantity of urea as dependent upon the accidental quantity of albumen in the blood.

An unprejudiced consideration of the researches which have hitherto been instituted for the purpose of deciding these questions, will at once show that they are altogether unsatisfactory. For on the one hand the chemical methods adopted for the quantitative estimation of urea were either liable to inaccuracy, or involved troublesome and tedious operations, which were applicable only in a few particular instances. On the other hand, it was certain that the constitution of urine and the quantitative relation of its several constituents are variably influenced by so many circumstances, that a correct insight into its qualitative importance and quantitative excretion could only be attained from a very great number of observations, and when the conditions under which they were made were exceedingly varied, and at the same time well known and definite.

If, therefore, the relation of urea to the general functions of life are to be more exactly investigated, and if the quantity in which it is excreted is to be recognized as the measure of metamorphosis of nitrogenous constituents of the organism, a method must be found for its quantitative estimation which will be at once certain, facile, and rapid in its execution.

Such a method has been contrived by Professor Leibig, which, with a little practice, admits of an estimation of urea being made in a quarter of an hour. I have in this manner instituted a large number of experiments with human urine and that of dogs and rabbits. The quantity of urea that the dog formed under the most diverse conditions of feeding was daily estimated during a whole year. The same was done for a period of five months with a rabbit.

For the human organism I have only endeavored to ascertain the quantitative relations of urea under the normal circumstances of life during long periods and for individuals of different sex and age. The results which have thus been obtained present very considerable discrepancies with the statements previously made.

In the case of the animals mentioned, however, I have more especially convinced myself that the determining conditions for the formation and excretion of urea are far more variable and multiform than has hitherto been supposed, and that they are influenced by circumstances so numerous and changeable that there is still a necessity for a much larger accumulation of accurate observations before the laws of this excretion and its corelative phenomena can be definitely evolved.

Although at present we can only consider the first step as having been taken, I believe that I have obtained some results which, while they remove previous doubt and present the subject under new aspects, may perhaps serve as the basis of further research.

Among these results are the following:

1. Urea is unquestionably, under all circumstances, the measure of the metamorphosis of nitrogenous constituents of the organism. It never originates from a direct metamorphosis of the albumen of the blood and vascular system. It is formed in the blood only from gelatin, and this perhaps never enters the blood unaltered in the normal conditions of life. The urea in this case is not a product of the metamorphosis of silid portions of the organism.

2. But although urea always originates in this manner from the metamorphosis in the organs, still, the quantity and quality of the food exercise a far greater influence upon the production of urea and the general metamorphosis than could hitherto have been supposed. It is indeed true that urea is formed and excreted under a total deprivation of food; but the per centage of nitrogen in the food exercises so great an influence upon the quantity, that when, for example, the dog on which I made my observations consumed in twenty-four hours 4000 grms. of cow flesh without fat or bone, he excreted in the same time 190 grms. of urea, while with 500 grms. of potato and 250 grms. of fat the quantity excreted was only 6 or 8 grms.

Food destitute of nitrogen, such as fat, under all circumstances produces a limitation of the metamorphosis of the nitrogenous portions of the organism. At the same time there is in most instances, *cæteris paribus*, a diminution in the quantity of urea excreted, but not always. When the food consists solely of fat both consequences obtain; the excretion of urea as well as the metamorphosis is diminished. The same is the case with a very full flesh diet.—With a flesh diet merely sufficient for maintaining the weight of the body fat limits the metamorphosis, but the quantity of urea excreted is not necessarily diminished at the same time; it may indeed become greater than that excreted when the same quantity of flesh is consumed without fat, in accordance with a law stated subsequently.

3. It has moreover been found, that the quantity of nitrogen in the food or portions of the organism metamorphosed within a certain period never appears entirely as urea, but that a certain, and under some circumstances considerable part, must be excreted in another form. This is likewise true in the case of dogs, although their urine does not contain uric acid, and scarcely a trace of other nitrogenous organic substances. Only very small quantities of nitrogen are excreted in the fauces, and as this is also true with regard to the lungs and skin, according to the admirable researches of Regnault and Reiset, it is difficult to form a correct opinion as to the form in which that part of the nitrogen of metamorphosed portions of the organism that is not found in the urine is excreted. It is most probable that this deficiency is owing to a partial conversion of urea in the blood, or perhaps even in the bladder, into carbonate of ammonia, which is excreted either by the skin and lungs or in the urine. However worthy of confidence the observations of Regnault and Reiset may be, I am still of opinion, that it has not hitherto been possible to continue them for a sufficiently long period, and under the necessary alterations of diet for determining with absolute certainty, whether or not carbonate of ammonia is excreted by the skin and lungs. The presence of carbonate of ammonia in the urine would be very probable, at least when, even with an exclusively flesh diet, or under a deprivation of food, it was alkaline while quite fresh and effervesced on the addition of an acid.

The quantity of nitrogen of the metamorphosed portions of the organism, which does not make its appearance as urea, is upon the whole tolerably constant under very diverse circumstances of diet and metamorphosis. It was found greatest, both relatively and absolutely, under a deficient supply of nitrogenous food (250 grms. of flesh.) It might in this case amount to more than two-thirds of the total nitrogen of the metamorphosed tissues. With a supply of nitrogenous food adequate for maintaining the weight of the body (500 grms. of flesh) it amounted to one-third. Under a very full and excessive flesh diet it was smaller absolutely than in the above cases, and was consequently so much reduced, relatively, as to be almost insignificant. I regard these facts as the strongest evidence that the original product of metamorphosis of nitrogenous tissues is solely urea, of which a certain portion experiences a further change —into carbonate of ammonia—proportionately greater when the quantity of urea is large than when it is small. The presence of fat in the food appears under certain circumstances to prevent or limit this further alteration of urea. It is owing to this influence, that although fat, as already remarked, limits the metamorphosis, and consequently the formation of urea upon the whole, still under a diet consisting of flesh and fat, the quantity of urea excreted may become greater than when the same quantity of flesh is taken without fat, because the nitrogen of the metamorphosed tissues remains in the form urea. I am of opinion that fat exerts this influence by virtue of its connexion with the process of respiration. Lastly, water exercises an influence upon the deficiency of nitrogen appearing as urea. Thus, for instance:

4. The quantities of water and urea always bear a very constant relation to each other. No other constituent of the urine has so decided an influence upon its density as urea. Dense urine always contains much urea; specifically light urine is always poor in urea. Nevertheless, the quantity of urea excreted upon the whole within a given period, is related in the most intimate manner with the quantity of water, and *cæteris paribus* a large quantity of urine carries off more urea than a small quantity passed in the same time, although its specific gravity may fall considerably at the same time.

This influence of water may be owing to several circumstances —an increased facility in the solution and extraction of urea; perhaps also to an increased facility in the formation of urea. But it is moreover quite certain that water has an influence upon the quantity of urea in so far as the time and rapidity with which the urine is evacuated depend upon its greater or less quantity. In the presence of much water the urea formed is very rapidly separated from the blood and from the organism. There is not much time then for any further alteration of the urea and consequently its quantity is greater while the quantity of nitrogen not in the form of urea becomes less. Hence it is more particularly explicable why with different quantities of nitrogenous food (flesh;) with little there is a comparatively and even absolutely great deficiency of nitrogen in the state of urea, and with much flesh, on the contrary, little deficit. For in the former case the quantity of urine passed is very small, often only a few cubic centimeters during several days; in the latter, on the contrary, very great, amounting to 1200 or 1500 cubic centimeters in twenty-four hours.

It follows from these facts, perhaps with certainty, that the quantity of urea excreted under certain circumstances and within a certain time, cannot be taken as the direct measure of metamorphosis in the tissues, even when the urine does not contain any other nitrogenous constituent. Still it will always be the most important element for ascertaining its amount, and it will only be necessary to study more closely the influences exerted upon its formation and excretion, towards the elimination of which I hope to have furnished some contribution.—*London Pharm. Journ.*, from *Annalen der Chemie und Pharmacie.*

